# The actual and anticipated effects of restrictions on flavoured electronic nicotine delivery systems: a scoping review

**DOI:** 10.1186/s12889-022-14440-x

**Published:** 2022-11-19

**Authors:** Christopher J. Cadham, Alex C. Liber, Luz María Sánchez-Romero, Mona Issabakhsh, Kenneth E. Warner, Rafael Meza, David T. Levy

**Affiliations:** 1 Department of Health Management and Policy, University of Michigan, School of Public Health, 1415 Washington Heights, Ann Arbor, MI USA; 2 Cancer Prevention and Control Program, Georgetown University-Lombardi Comprehensive Cancer Center, 3300 Whitehaven St, Washington, DC USA; 3 Department of Epidemiology, University of Michigan, 1415 Washington Heights, Ann Arbor, MI 48109-2013 USA

**Keywords:** Electronic nicotine delivery system, Flavours, Flavoured e-cigarette, Flavoured tobacco, Restriction, Tobacco regulatory science, Tobacco regulation, Scoping review

## Abstract

**Objective:**

To synthesize the outcomes of policy evaluations of flavoured electronic nicotine delivery systems (ENDS) restrictions.

**Data sources:**

PubMed, Scopus, Embase and Web of Science before May 3, 2022.

**Study selection:**

Studies that report sales, behaviour, or compliance outcomes related to implemented or hypothetical ENDS flavour restrictions.

**Data extraction:**

Restriction details, whether implemented or hypothetical, whether additional products were restricted, jurisdictional level, study locations, and outcomes classified by sales, behaviour, and compliance.

**Data synthesis:**

We included 30 studies. Of those, 26 were conducted exclusively in the US, two in India, and two surveyed respondents in multiple countries, including the US. Twenty-one evaluated implemented restrictions, while nine considered hypothetical restrictions. Five studies evaluated product sales, 17 evaluated behaviour, and 10 evaluated compliance, with two studies reporting multiple outcomes. Two studies reported an increase and one a reduction in cigarette sales following restrictions, while three reported reductions in ENDS sales. Behavioural studies presented a mixed view of the impacts of regulations on ENDS and cigarette use. However, the use of disparate outcomes limits the comparability of studies. Studies of hypothetical restrictions suggest decreased ENDS use, increased cigarette use, and increased use of illicit markets. Studies of compliance with flavoured product restrictions that included ENDS found that 6–39% of stores sold restricted flavoured products post-restrictions. Online stores remain a potential source of restricted products.

**Conclusion:**

Our findings highlight the need for additional research on the impacts of ENDS restrictions. Research should further evaluate the impact of restrictions on youth and adult use of nicotine and tobacco products in addition to the effects of restrictions in countries beyond the US to enable a robust consideration of the harm-benefit trade-off of restrictions.

**Supplementary Information:**

The online version contains supplementary material available at 10.1186/s12889-022-14440-x.

## Introduction

Electronic nicotine delivery systems (ENDS) come in hundreds of flavours beyond tobacco [1]. These non-tobacco flavours (henceforth flavoured ENDS) are thought to appeal to youth, leading to increased ENDS initiation, thereby making flavours a potential driving factor in the youth vaping epidemic [2]. As a result, there is growing concern that the widespread availability of flavours may lead a new generation to nicotine addiction when youth tobacco use rates are at an all-time low [3, 4]. These concerns were exacerbated by the emergence of e-cigarette or vaping use-associated lung injury (EVALI) in 2019, following which eight US states imposed temporary restrictions on flavours in ENDS [5]. In 2020, seven states passed permanent restrictions on flavoured ENDS [5]. Locally, over 300 jurisdictions have imposed restrictions on flavoured ENDS, with the majority (168) in Massachusetts before their statewide ban [6, 7]. The restrictions vary substantially, with adult-only establishments exempt in over half of local restrictions [8]. ENDS flavour restrictions have also proliferated internationally, with at least nine countries restricting the sale of flavoured ENDS in some way [9].

In 2018, the US Food and Drug Administration (FDA) published an advanced notice of proposed rulemaking restricting flavours other than tobacco in ENDS, along with a ban on menthol in cigarettes and all flavours in cigars [10]. The bans on combustible flavoured products were formally proposed in April 2022 [11]. Additionally, the FDA has issued marketing denial orders for millions of ENDS products through its Premarket Tobacco Product Application process, citing applicants’ failure to demonstrate that approval would be appropriate for the protection of public health [12, 13]. While the agency has approved a small number of tobacco-flavoured ENDS applications, it has yet to approve a flavoured ENDS application [14].

Flavour restrictions may successfully reduce ENDS initiation and remove a potential gateway to combustible tobacco use among youth [2]. They may also reduce potential harm reduction benefits as fewer current adult smokers switch to ENDS, hindering their efforts to quit smoking [15]. The overall public health impact of these restrictions depends on the balance of never smokers or former smokers who initiate tobacco use due to ENDS availability vs. current smokers who quit or switch to exclusive ENDS use [16]. Concerns over long-term use of either product should be based on the relative harm of the products [17, 18]. Flavour restrictions may also lead to the rise of illicit products or do-it-yourself ENDS flavouring, with potential new health risks [19, 20]. The impact of a restriction is likely to depend on the products covered, flavours that remain available, the exemption of adult-only establishments, the availability of products at retailers within and outside a jurisdiction, and enforcement efforts [21].

To inform the debate over national policy and future studies of the effects of these policies, [22] we synthesize the current literature on implemented or hypothetical restrictions on product sales that include flavoured ENDS. Given the novelty of flavoured ENDS restrictions and the limited availability of data, we opted to conduct a scoping review to identify the current evidence on the impacts of restrictions and ongoing knowledge gaps with the goal of providing an overview of the evidence [23]. We provide detailed descriptions of study methods and outcomes to facilitate the identification of limitations of the body of evidence.

## Methods

This review follows the Preferred Reporting for Systematic Review and Meta-Analysis Extension for Scoping Reviews (PRISMA-ScR) guidelines [24]. The review was not registered. However, a prespecified protocol and details on deviations from the protocol are available in Appendix 1 and the PRISMA-ScR checklist in Appendix 2.

### Data sources and searches

We searched Medline (PubMed), Scopus, Embase and Web of Science for articles relating to the effects of restricting flavoured ENDS on tobacco product sales, self-reported behaviour, and compliance. The complete strategy is detailed in Appendix 3. No date, study type, or geographic restrictions were used in the search term. We searched selected studies’ references to identify other potentially relevant studies. The last search was conducted on May 3, 2022.

### Study selection

The primary study team (CJC, ACL, DTL) agreed upon eligibility criteria prior to study screening*.* Given the scoping nature of this review, we included peer-reviewed studies that consider the effects of exposure to an implemented or hypothetical flavoured ENDS restriction. Flavour restrictions could exclude some flavours, such as mint or menthol (all restrictions that did not completely ban e-cigarettes permitted tobacco flavour). Studies with broader restrictions on products other than ENDS were included if flavoured ENDS were among the products restricted. Bans that exempted specific retailers, such as tobacconists, vape shops, or age-restricted establishments, were also included.

We excluded: non-peer-reviewed empirical studies; studies published in languages other than English; opinion articles; editorials; studies not reporting results specific to individual behaviour, sales, or compliance; and studies reporting on jurisdictions that did not yet have legal ENDS sales, as their findings would not relate to post-legalization restrictions.

One team member (CJC) reviewed titles and abstracts, and conducted the full-text screening using DistillerSR [25]. The primary study team resolved uncertainty over inclusion.

### Data abstraction

One team member (CJC) extracted the relevant information from the selected studies using DistillerSR, including information on the: restriction type, outcome type(s), sample size, age group, location, restriction implementation date, list of all banned products, the data source and study method, results, and study limitations. Extracted results were limited to sales of tobacco products, use of tobacco products (including ENDS, cigarettes, and other tobacco products), and compliance (such as the availability of flavoured products).

### Data synthesis

Following extraction, we categorized articles by type of outcome: sales, self-reported behaviour, or compliance. Within the behaviour category, we distinguished hypothetical studies from studies of implemented restrictions.

### Quality appraisal

Given substantial heterogeneity in research questions, methods, and outcomes of interest, we did not conduct a formal quality appraisal of individual studies. Following Rogers et al., [26] we use the Grading of Recommendations, Assessment, Development and Evaluation (GRADE) approach to evaluate the quality of the body of evidence presented in the research on flavour restrictions [27]. Instead of reviewing individual study quality, GRADE ratings and adjustments are based on factors contributing to the strength or weakness of a body of evidence on a given outcome. GRADE uses four confidence levels from Very Low to High, reflecting the confidence that an outcome is accurate based on the current literature. Additional details on the GRADE approach used here are presented in Appendix 4.

## Results

**Fig. 1:**
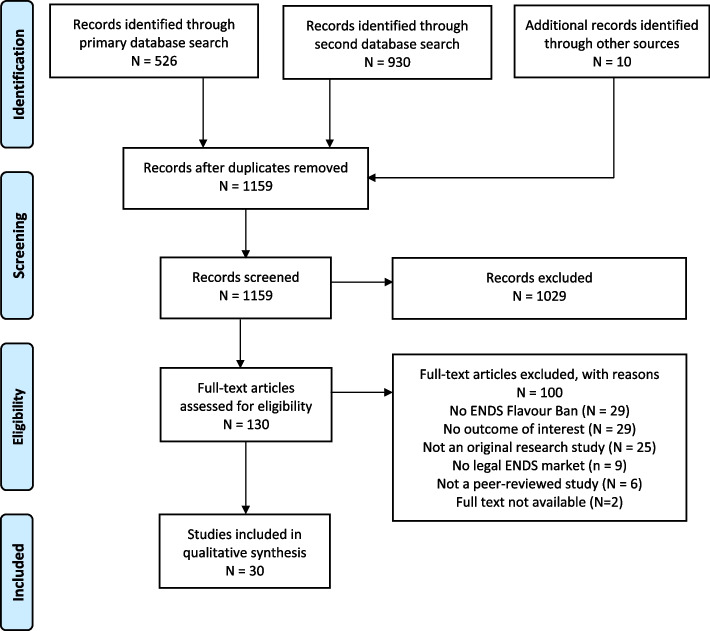
Preferred Reporting Items for Systematic reviews and Meta-Analyses flow diagram of study selection

We identified 1159 unique records; 30 were deemed eligible for inclusion. The PRISMA diagram is presented in Fig. 1.

Table 1 presents the breakdown of studies by restriction type, outcome, and location. Appendices 5–7 provide detailed descriptions of study methods and results. Nine studies produced results related to hypothetical restrictions, and 21 examined implemented ones. Thirteen studies examined restrictions exclusive to ENDS (including flavour restrictions and total ENDS restrictions), while 19 examined restrictions including ENDS but extended to other flavoured products; two hypothetical studies reported on both types of restriction. Most studies examine local jurisdiction restrictions, either at the town, city or county level, all located in the US. Below, we summarize key findings from studies organized by outcome.

**Table 1:** Characteristics of included studies

**Study Type**^**a**^				
***Restriction Type***	***Sales***	***Behaviour - Implemented***	***Behaviour - Hypothetical***	***Compliance***
Total ENDS Ban	4	1	2	3
Flavoured ENDS Restriction	3	1	6	0
ENDS Restriction Exempts Some Flavours	0	0	4	2
ENDS Restriction Exempts Some Retailers	0	3	0	3
All Flavoured Tobacco Product Ban	1	4	0	5
**Location**^**a**^				
***Restriction Type***	***US - Local***	***US - State***	***US - National***^**b**^	***Non-US***
Total ENDS Ban	1	5	1	2
Flavoured ENDS Restriction	1	5	4	2
ENDS Restriction Exempts Some Flavours	2	0	4	0
ENDS Restriction Exempts Some Retailers	6	0	0	0
All Flavoured Tobacco Product Ban	9	0	0	0
**Age Group**^**c**^				
Youth	6			
Young Adults	3			
Adults	8			
**Industry Sponsored**				
Yes	1			
No	29			

^a^ Studies included multiple types of restrictions. Counts of restriction types sum to greater than the included number of studies

^b^ Studies were exclusive to hypothetical restrictions

^c^ Only studies with behaviour outcomes included

### Sales

Five US-based studies evaluated sales of nicotine and tobacco products following adoption of flavour restrictions using retail scanner data that is passively collected, aggregated and sold to researchers by the firms Nielsen and IRI (Table 2; Appendix 5). Three examined the effects of policies across multiple states, one in a single state and one in a local jurisdiction.

**Table 2:** Summary of tobacco product sales results

	All Tobacco Products	ENDS	Cigarettes	OTP^a^
Sales Rise	1	1	2	0
Sales Flat	0	0	1	0
Sales Fall	0	3	1	1
Not Reported	4	1	1	4

^a^ OTP – other tobacco products

### Local restrictions

In California, Gammon et al. [28] compared average weekly sales of tobacco products from 2015 to 2019 in San Francisco (SF), San Jose (SJ), and San Diego (SD), before and after SF’s restriction on all flavoured products. They report that SF saw a 25% decrease in total product sales following the restriction, compared to 8 and 17% in SJ and SD, respectively. Except for cigar sales in SJ, declining sales were reported in all regions for total cigarette (− 23, − 13%, − 22%), cigar (− 51, 1, − 13%), and smokeless (− 37, − 10%, − 4%) sales for SF, SJ, and SD, respectively. The three cities saw increases in total ENDS sales (SF: 44%, SJ: 171%, SD: 98%).

### Statewide restrictions

Katchmar et al. [29] used a mixed-methods approach that included the analysis of resident surveys (see behaviour section) and Nielsen sales data using interrupted time-series to evaluate the impact of changes to Massachusetts’ tobacco product regulations. In response to the September 2019 EVALI outbreak, Massachusetts imposed a temporary ban on all ENDS sales that became a permanent restriction on flavoured ENDS in December 2019. In addition, an excise tax was imposed on ENDS in June 2020. The authors report that the greater Boston area, including areas in other states (Rhode Island and southern New Hampshire) not covered by the restriction, saw a significant decrease in ENDS sales compared to the remaining US following the temporary ENDS ban. However, sales in Boston levelled off over time as US sales continued to fall. They report no change in cigarette sales following the initial temporary ban on ENDS. Following the switch to a permanent flavour restriction, cigarette sales did not change significantly in either Boston or the US compared to the previous year, but relative to the US, Boston saw a statistically significant increase in the rate ratio of cigarette sales.

The three remaining sales studies used multivariate approaches that considered results from non-flavour ban jurisdictions as comparators to evaluate the impact of restrictions across multiple states. Liber et al. [30] examined temporary ENDS flavour restrictions in Michigan, Oregon, and Washington following the EVALI outbreak and the temporary restriction on all ENDS sales in Massachusetts. Using Nielsen sales data, they found that ENDS sales fell more in states with flavour restrictions and Massachusetts than in states with no restrictions. However, only the decline in Massachusetts was statistically significant. Additionally, the total ban in Massachusetts was associated with a statistically significant increase in cigarette sales. No significant change in cigarette sales was observed in states with an ENDS flavour restriction.

Ali et al. [31] used a Difference-in-Differences (DiD) [32] design to evaluate the impact of restrictions in Massachusetts, New York, Rhode Island, and Washington on ENDS sales using IRI retail scanner data. Compared to 39 control states, the Massachusetts restriction on all ENDS was associated with a statistically significant 94.4% decrease in mean 4-week ENDS sales. The narrowing of the restriction to only flavoured ENDS was associated with an 88.9% reduction in mean 4-week ENDS sales. In New York, Rhode Island, and Washington, overall mean 4-week ENDS sales fell by 25.0–31.3%.

Finally, a group of researchers from JUUL used a DiD design and IRI data to evaluate the impact of the 2019 restrictions in Massachusetts, Rhode Island, and Washington on per capita cigarette sales [33]. They found that Massachusetts’s complete ENDS ban significantly increased cigarette sales. Their preferred model obtained a 7.5% increase in cigarette sales in Massachusetts. The flavour restrictions in Rhode Island and Washington were associated with increases in cigarette sales of 1.0 and 8.7%, respectively, with only the latter statistically significant.

### GRADE score

We evaluated the quality of evidence regarding reduced sales of ENDS and increased sales of combustible cigarettes (Table 3). The quality of evidence supporting ENDS sales reductions was deemed **moderate** given the consistent and large magnitude of effects seen (three of four studies found declines) and a potential dose-response relationship between the extent of the ENDS products banned and the degree to which an effect was seen (complete ban on ENDS vs. flavours only). Concerns over limitations in study design and potential publication bias downgraded the findings. The quality of evidence supporting the increased sale of combustible cigarettes was deemed **low** due to inconsistent findings across studies (two studies found an increase, one no change, and one a decrease).

**Table 3:** Quality of Evidence of Outcomes of Restrictions on the Sale of Flavoured ENDS

Outcome	Quality of Evidence	Factors that Increase Quality	Factors that Reduce Quality	Supporting Evidence
**Sale**				
Reduced sales of ENDS	Moderate	Dose-response gradient (ban on all ENDS vs. flavours) (+ 1) Large magnitude of effect (+ 2)	Limitations in study design (− 1) Potential publication bias (− 1)	Ali (2022), Gammon (2021), Katchmar (2021), Liber (2021) [28–31]
Increased sales of combustible cigarettes	Low	Dose-response gradient (ban on all ENDS vs. flavours) (+ 1) Moderate magnitude of effect (+ 1)	Inconsistent findings (− 1) Limitations in study design (− 1)	Gammon (2021), Katchmar (2021), Liber (2021), Xu (2022) [28–30, 33]
**Behaviour**				
Reduced consumption of any tobacco use	Low	Moderate effect sizes across studies (+ 1)	Inconsistencies in measurement rigour (differing definitions of product use) (− 1) Low measurement rigour (− 1)	Kingsley (2019, 2021), Olsen (2022), Yang (2022) [38–41]
Reduced ENDS consumption	Low	Moderate effect sizes across studies (+ 1)	Inconsistencies in measurement rigour (differing definitions of product use) (− 1) Low measurement rigour (− 1)	Hawkins (2021), Kingsley (2019), Liu (2022), Yang (2020) [35, 38, 39, 42]
Increased combustible cigarette consumption	Very Low	Large magnitude of effect (+ 2)	Limitations in study design (− 1) Inconsistency in controls for bias risk (− 1) Inconsistent findings (− 1) Low measurement rigour (differing definitions of product use) (− 1)	Friedman (2021), Hawkins (2021), Kingsley (2019), Liu (2022), Yang (2022) [34, 35, 38, 39, 42]
**Compliance**				
Reduced availability of flavoured products	Moderate	Dose-response gradient (difference in enforcement) (+ 1) Large magnitude of effect across studies (+ 2)	Limitations in study design (−1) Inconsistencies in measurement rigor (short-term follow-up) (− 1)	Amalia (2020, 2020), Andersen-Rodgers (2021), Brock (2019), Gaiha (2021), Holmes (2022), Kephart (2020), Kingsley (2019), Nali (2021), Vyas (2021) [39, 53–61]

The numbers in brackets refer to the extent to which GRADE factors upgraded or downgraded our confidence in the quality of evidence

### Behaviour

We identified 17 studies that reported the effects of flavour restrictions on self-reported tobacco use behaviours (Appendix 6). Eight studies examined implemented restrictions in the US, and nine examined hypothetical restrictions.

### Implemented restrictions

Seven studies examined local jurisdiction restrictions and one statewide. Seven studies examined youth, one young adults, and one adults.

### Local restrictions

In SF, CA, Friedman [34] used a DiD approach to quantify the impact of the flavoured tobacco product ban (including ENDS) on past 30-day tobacco use among high school students using 2019 Youth Risk Behavior Surveillance System data. She found that the ban was associated with a doubled odds of recent cigarette smoking among high school students relative to control districts (adjusted odds ratio: 2.24). A critique [35] argued that the data used in the study was collected prior to enforcement of the restriction and, as such, considered it an inaccurate representation of the restriction’s impacts. Instead, the authors pointed to neighbouring Oakland, CA, which saw a purported decline in high school youth vaping and cigarette use from 2017 to 2019 following the July 2018 ban on convenience store flavoured ENDS sales. However, the authors conducted no formal analysis of their own. In response, Friedman’s study examined the effective date of the restriction rather than the enforced date and still found the reported associations [36, 37]. Yang et al. [38] also evaluated the SF, CA restriction using a survey of 247 young adults ages 18–34. They found a lower prevalence of any tobacco product use and any flavoured product use after the ban compared to pre-restriction behaviour. They reported a non-statistically significant increase in cigarette smoking prevalence among those ages 18–24.

In Massachusetts, Kingsley et al. [39] conducted a DiD analysis of the impact of a June 2016 flavoured tobacco ban in the city of Lowell on high school-aged youth nicotine and tobacco use behaviours, with the city of Malden serving as a control. There were no significant differences in the change in the likelihood from baseline to follow-up that a student would initiate tobacco use with a flavoured product. However, the authors found larger declines in ever-use (− 6.1%) and current use (− 5.7%) of any flavoured tobacco product in Lowell compared to Malden, with only the latter statistically significant. Additionally, there was significantly lower ever-use and current use of any non-flavoured tobacco product.

Again using DiD, Kingsley et al. [40] evaluated the impact of flavoured tobacco restrictions on high school student tobacco use in the cities of Salem and Attleboro compared to Gloucester, MA. They report that ever and current use of flavoured and non-flavoured or menthol tobacco increased in all three cities from baseline to follow-up, but current use of flavoured and non-flavoured or menthol tobacco was significantly smaller in adopting municipalities. They also conducted focus groups where some respondents discussed leaving the county or state to purchase products [40]. Olsen et al. [41] found similar effects in Minnesota, where any tobacco product use among youths remained flat following the 2016 ban on any flavoured products in Minneapolis and St. Paul but rose in the rest of the state. ENDS use in the restricted cities rose over the period but significantly less than in the rest of the state.

Hawkins et al. [42] used DiD to determine the effects of county-level restrictions on all flavoured tobacco products not sold in adult-only establishments on days of cigarette use and ever use of ENDS among high school students in Massachusetts from 2011 to 2017. Using the Massachusetts Youth Health Survey, they found that increasing tobacco restrictions were not associated with a reduction in the likelihood of past 30-day cigarette use but with a decrease in the number of days of cigarette use. Additionally, restrictions were associated with a significant reduction in ENDS use (adjusted odds ratio: − 0.87).

### Statewide restrictions

In addition to their Nielsen sales data analysis, Katchmar et al. [29] surveyed Massachusetts residents about tobacco use. In a poorly powered survey of 36 adult residents, the authors found no change in daily ENDS use after policy implementation. Additionally, there was an increase in the number of respondents who indicated that they made trips to other states to purchase ENDS.

### Hypothetical restrictions

Four hypothetical restriction studies evaluated the impacts of a ban using discrete choice experiments (DCEs), [43] four conducted surveys, and one used interviews. Seven studies surveyed only US residents, while two surveyed residents of multiple countries, including the US. All but two asked about bans exclusive to flavoured ENDS. Two studies evaluated the potential impacts of restrictions on youth and young adult behaviour, while the remaining seven considered adult behaviour.

In their DCE, Pesko et al. [44] evaluated the impacts of flavour restrictions and warning labels on tobacco product selection in a purchase experiment. Results from this study were later analyzed to consider impacts on product switching [45]. Results from the DCE with 1200 adult smokers indicated that increased flavour availability significantly increased ENDS selection for young adults from 17.5 to 21.9% but not older smokers. Additionally, greater flavour availability increased ENDS selection for individuals who had not used vaping devices in the past month. The subsequent evaluation of different policy scenarios using the results from their DCE found minimal evidence of any changes in ENDS or cigarette use [45].

Buckell et al. [46] compared five alternative policies: ban all flavoured tobacco products; allow only tobacco and menthol ENDS; ban all cigarette flavours; allow only fruit/sweet ENDS; and ban all ENDS flavours. The 2031 adult smokers in the study selected their preferred products under each restriction scenario in a virtual marketplace. They report that restricting all flavoured ENDS alone would likely increase cigarette use (8.3% increase in cigarette selection). Additionally, a restriction on all flavoured tobacco products would reduce ENDS use (7.9% decrease in ENDS selection) with the greatest increase in ‘opting-out’ (5.2% increase in no selection), but with a 2.3% increase in cigarette selection.

In the final DCE, Freitas-Lemos et al. [47] sought to evaluate the role of flavour bans and price increases on the illicit tobacco market. A sample of 150 adult smokers, ENDS users, and dual users considered three scenarios: no restriction; a restriction on flavoured ENDS; and a ban on all ENDS. Respondents then made choices in an experimental marketplace under each restriction and given the option of purchasing banned products through an illicit experimental marketplace. Restrictions increased the likelihood of purchasing from the illicit market. A complete vaping ban had greater impact than only flavour restrictions. Additionally, willingness to purchase from the illicit market was highest among exclusive ENDS users.

Farsalinos et al. [48] asked a global sample of 4618 ENDS users: “how would your experience with EC [e-cigarettes] change if flavours variability was limited?” They found that under a flavour restriction, 68.9% of respondents would find ENDS less enjoyable; 45.7% would find ENDS more boring; 48.5% would have increased cravings for cigarettes; 39.7% would be less likely to reduce or quit smoking (44.2% among current cigarette smokers vs. 39.3% among former cigarette smokers), and 6.3% indicated no difference.

Gravely et al. [49] surveyed 851 ENDS users in Canada, England and the US about proposed flavour restrictions on ENDS. Of the 703 who stated their intentions following a hypothetical ban on flavours, 28.8% would plan to continue vaping with an available flavour, 28.3% would find a way to get banned flavours, 17.1% would stop vaping and smoke cigarettes instead, 12.9% said that they would stop vaping and not smoke, and 12.9% did not know what they would do. Statistically significant differences were seen among dual-users, who were more likely to become exclusive smokers, and women, who were more likely to report plans to find a way to get their preferred flavour or quit entirely.

Huh et al. [50] interviewed 276 adult vape shop customers. They used structural equation modelling to evaluate the interrelationship between product preference, e-cigarette dependence, e-cigarette harm perception, and purchase/use intention, given a hypothetical flavour ban. They found that those who preferred non-tobacco flavours showed significantly lower intentions of continued purchase and use of e-cigarettes with a flavour restriction. Those who reported using vaping to quit cigarettes indicated greater intentions for continued purchase and use of e-cigarettes.

In their survey, Posner et al. [51] asked 2159 young adults, 550 of which were past 30-day ENDS users, in six US metropolitan areas their likelihood of using ENDS or cigarettes following a flavoured ENDS restriction and their likelihood of switching to cigarettes following a complete ENDS restriction. With a flavour restriction, 39.1% of ENDS users indicated being “very or somewhat likely” to continue using ENDS, 33.2% reported being “very or somewhat likely” to switch to traditional cigarettes, and 14.9% reported being “very or somewhat likely” to become dual users. If all vaping products were restricted, more users reported intentions to use cigarettes (39.4%). Among exclusive ENDS users, 72.2% reported being “not at all likely” (39.4%) or “a little likely” (32.8%) to continue vaping if vape product flavours were restricted, while 79.8% reported being “not at all likely” (72.7%) or “a little likely” (7.1%) to switch to cigarettes if vape product flavours were restricted. The remaining 21.2% reported being “somewhat likely” or “very likely” to switch to cigarettes.

Finally, Pacek et al. [52] surveyed 240 young adult dual ENDS and cigarette users on their expected product use following an ENDS flavour restriction and other ENDS regulations. Respondents were asked if they expected to increase, maintain, reduce, or quit ENDS and cigarette use for each policy. They found that among users of flavoured e-liquids, participants were likelier to indicate that they would quit or reduce their use of ENDS and maintain or increase cigarette over ENDS use. As a result of a flavour ban, 12 and 5% of all users reported the intention to quit ENDS and cigarettes, respectively. Reduction of product use was 42% for ENDS and 25% for cigarettes, while 40 and 54% would maintain ENDS and cigarette use, respectively. Only 5 and 18% would increase ENDS and cigarette use, respectively.

### GRADE score

We evaluated the quality of evidence from studies of implemented restrictions on three behaviour outcomes: reduced any tobacco product use, reduced ENDS consumption, and increased combustible cigarette consumption (Table 3). Hypothetical studies were excluded from the quality assessment as they only provided indirect evidence of behavioural intentions. The quality of evidence regarding any tobacco use and ENDS consumption was considered **low** given the moderate effect sizes, measures of self-reported product use, and differing definitions of product use (current vs. ever). Despite some large effect sizes, evidence regarding increased combustible cigarette consumption was considered **very low** given the inconsistencies in study findings, study design limitations and potential risks for bias.

### Compliance

Ten studies reported compliance issues following implemented flavoured ENDS bans (Appendix 7). In two, the bans were exclusive to flavoured ENDS, while the remaining eight included other flavoured tobacco products. Eight studies were from the US, seven evaluated local restrictions and one state restriction, and two were from India.

### Local restrictions

Using San Francisco Public Health Department data on retailer inspections from December 2018 to December 2019, Vyas et al. [53] reported that following the start of enforcement, 77–100% of licenced tobacco retailers did not sell the restricted products (i.e., all flavoured nicotine or tobacco products). Compliance peaked near the start of enforcement in April 2019. The high compliance is further supported by Holmes et al. [54] They conducted retail store visits in San Francisco and cities in Alameda county in 2015 and 2019–2020 to compare cities with full or partial flavour restrictions to those without restrictions. In their pre-post analysis, they report a statistically significant decrease in the availability of Blu menthol e-cigarettes, the one ENDS they recorded in their pre-restriction visits, following the adoption of restrictions compared to non-restricted cities. They also found lower post-restriction availability of flavoured ENDS in restriction cities than in those without restrictions (21.3% vs. 86.6%).

Gaiha et al. [55] conducted an online survey of 15–29 year-olds in California to compare the odds of purchasing flavoured products at retail stores in regions with and without restrictions. They found that underage ENDS users in jurisdictions with restrictions had significantly lower odds of retail purchases. Additionally, the restrictions did not increase the likelihood of online sales access but did increase the odds of social access. Andersen-Rodgers et al. [56] also compared local California jurisdictions to determine their impact on flavoured product availability. From observations of 325 stores, a significantly lower proportion of stores in flavour ordinance jurisdictions compared to those in jurisdictions without restrictions sold menthol cigarettes (40.6% vs. 95.0%), cigarillos/cigar wraps with explicit flavour names (56.4% vs. 85.0%) and ENDS with explicit flavour names (6.1% vs. 56.9%).

In Massachusetts, Kingsley et al. [39] found that following the flavoured tobacco product restriction in Lowell, the sale of flavoured products decreased significantly from pre-restriction (September 2016) to 6-months after implementation (March 2017), with a 70% reduction in stores where flavoured products were available. They found no change in the control city over the same period. Kephart et al. [57] evaluated compliance with Boston’s local ban on all flavoured tobacco products by conducting retail observations between January and December 2016, with 488 retailers at baseline and 469 retailers eight months after the policy implementation. At baseline, 88.6% of retailers sold flavoured tobacco products, while at follow-up, 14.4% sold flavoured products. The number of e-cigarette/liquid products available decreased from 1135 to 17. Of the 51 retailers not in compliance, 72.5% reported not knowing a product was in violation.

The final local compliance study examined the retail availability of flavoured products in Minneapolis and Saint Paul, MN, following the restriction on all flavoured tobacco products except by tobacco-specific retailers. Brock et al. [58] conducted retail observations at 92 stores before and after implementation and found that significantly fewer convenience/grocery stores sold flavoured tobacco in Minneapolis (85.4% vs. 39.0%) and Saint Paul (97.3% vs. 8.1%). The study did not differentiate by types of flavoured products still available.

### Statewide restrictions

Nali et al. [59] evaluated the compliance of online retailers with the Massachusetts restriction on flavoured ENDS by attempting to purchase flavoured ENDS from 50 online vendors. Of these sites, 80% used some form of age verification, and 76% were non-compliant with the statewide restriction. Follow-up compliance checks found that only 28–30% of online retailers were compliant.

### National Restrictions - India

In India, 18.6% of a sample of 199 retailers still sold ENDS following a complete ENDS prohibition [60]. Most non-compliant retailers were tobacco retailers (94.6%). Of the stores that still sold ENDS following the restriction, 90% were aware the products were banned. A second study in India looked at the availability of ENDS online post-restriction [61]. They found 45 unique websites, 35.6% of which delivered at least one vaping product to New Delhi. Half of these non-compliant websites were general e-commerce sites; 10 were from other countries.

### GRADE score

We evaluated the quality of the evidence of one compliance outcome: reduced availability of flavoured products (Table 3). The GRADE score for this outcome was **moderate,** given the large reductions in flavour product availability in stores following restrictions and the gradient of effect seen with differing levels of enforcement. These factors are mitigated by short-term follow-up and the lack of consideration for online product availability, which was found to be less impacted by restrictions.

## Discussion

Our scoping review has identified limited information regarding the effects of bans or restrictions on flavoured ENDS products. While we identified 30 studies, the overall findings on the potential impact of a federal flavoured ENDS restriction are mixed and inconclusive, pointing to the need to evaluate these policies more systematically as additional jurisdictions impose them.

Of the five sales studies, one reported a reduction in cigarette sales, [28] one flat sales, [29] and two an increase [30, 33]. Three studies reported a decline in ENDS sales [29–31] and one an increase following the restrictions, although the increase was lower than observed in control regions [28]. One of the included studies of sales restrictions came from a group of researchers at JUUL, which raises concerns over potential bias given that tobacco industry-sponsored studies have been found to have more industry favourable results [62]. Sales of nicotine and tobacco products can provide an immediate indication of the impact of a policy. However, drawing concrete conclusions on patterns of use from this data is challenging, especially for smaller jurisdictions where individuals can easily travel to areas without restrictions and buy products online or at exempt retailers.

Turning to evaluations of implemented restrictions on individual behaviour, studies suggest that, following ENDS restrictions, youth ENDS and dual users may be more likely to be past 30-day cigarette users but use cigarettes less frequently or use fewer cigarettes. Results from local restrictions in Massachusetts suggest limited reductions in the likelihood of initiating tobacco use with flavoured products but reductions in the rate at which current use of any nicotine or tobacco product increases. Restrictions may also play a role in the frequency or intensity of use, as suggested by Hawkins et al.’s findings of reduced days of cigarette smoking [42]. Among these studies, the comparability of individual findings is unclear, given the variation in outcomes. Future studies should attempt to use similar measures and report related outcomes for set products across jurisdictions and age groups to aid comparability.

Hypothetical studies provide insights into smokers’ and ENDS users’ self-reported behavioural intentions in response to a variety of different restrictions. However, as studies of the link between quit intention and quit success have consistently demonstrated, there are limits to the extent to which behavioural intention leads to successful cessation of smoking [63–65]. Together, these studies suggest ENDS users would get less satisfaction from the products if flavours were banned. Flavoured ENDS restrictions could decrease ENDS use, while 17.1–33.2% of users might switch to combustible cigarettes [46, 49, 51, 52]. Gravely et al. [49] also indicate a large group of ENDS users who may try to evade restrictions.

Finally, the studies of compliance point to the importance of strong enforcement to ensure the success of restrictions. Overall, these studies suggest that, with enforcement in place, flavour restrictions substantially reduce the availability of flavoured products in mass-market retail stores. Retail observation studies found that following a ban, 6–39% of stores still sold flavoured products but with higher compliance for flavoured ENDS and e-liquids. These findings are consistent with research examining retailers’ compliance with other flavoured tobacco restrictions [26, 66]. Compliance studies also suggest additional challenges of regulating the online market, as observed in Massachusetts and India [59–61].

Our review points to several gaps in the current literature on ENDS flavour restrictions. Thus far, evaluations of implemented restrictions on individual behaviour have almost exclusively focused on youth and young adults, ignoring the potential impacts of flavour restrictions on adult smokers and vapers. Understanding the impact on both age groups is essential to determining the public health impacts of ENDS restrictions [18].

Additionally, it has been well documented that ENDS restrictions vary in their comprehensiveness [8]. How the comprehensiveness of the restriction impacts tobacco product use remains unclear. Some jurisdictions ban ENDS entirely, while others exempt certain flavours or types of retailers. Some studies of sales did directly compare the impact of total ENDS restrictions compared to flavours [30, 31, 33]. However, we did not find sufficient evidence to compare the impacts of bans that exempted certain flavours. Few studies of local restrictions [28, 34, 38, 42] and hypothetical studies [46] examined restrictions that included other tobacco products. These studies illustrate how the impacts of an ENDS flavour restriction may depend on other flavour restrictions, such as menthol in cigarettes. However, it remains hard to disentangle the effects from the identified studies and extrapolate to the population level [22, 67].

No study examined the differential impact of extending a restriction to all retailers rather than exempting ‘over 21’ establishments such as vape shops and the impact that restriction might have on combustible tobacco use compared to ENDS. Such a policy might better balance the needs of current smokers looking to quit and policymakers looking to avoid youth vaping [68, 69]. Flavour restrictions have become increasingly popular. However, taxes and educational campaigns have proven to be particularly effective at reducing youth tobacco product use in addition to reducing adult use [68, 69]. At a minimum, the unclear findings from our review illustrate that favour restriction should not be considered in isolation but as part of a complete policy agenda that looks to balance the need of current smokers and youth prevention.

Finally, there is an evident lack of research on ENDS flavour restrictions outside the US. While at least nine countries have restricted flavours, [9] we only identified four studies that looked beyond the US: two hypothetical studies that included samples in various countries and two studies of India’s restriction. Future research in this area is needed in countries like Canada and the Netherlands, which implemented restrictions in 2022. Corroboration of findings from the US in other countries would provide further support for the effects of such restrictions. At the same time, contrary studies may help indicate the factors (such as the exemption of specific flavours or retailers and different enforcement efforts) that make restrictions more or less successful.

### Limitations

This review is not without limitations. The policies evaluated, analytic methods used, and outcomes reported from the included studies are heterogeneous. The variation in outcomes, methods, and policies limits the approach of evidence synthesis tools like GRADE. Rather than use a set cut-off to determine whether groups of study findings had moderate or large effects, two authors discussed the findings to make a determination. This approach introduces subjectivity into the GRADE rankings. However, it does not detract from our overall conclusion that the effects of ENDS flavour restrictions remain uncertain. Additionally, current flavour bans or restrictions across jurisdictions have different rules that limit comparability [8]. A complete restriction on ENDS will likely have different effects than a restriction on only some flavoured products. Restrictions in smaller jurisdictions, like single cities or counties, are not comparable to state or national bans, partly because “border crossing” to obtain flavoured products from neighbouring jurisdictions is so easy. Travelling to a neighbouring state, in the case of a statewide ban, is generally more difficult. Smaller jurisdictions may also lack the resources for enforcement possessed by state or federal agencies. However, larger-scale restrictions may provoke more coordinated responses from manufacturers to produce an engineered solution around a flavouring restriction, as seen by promoting DIY flavouring additives for cigarettes in the EU [70, 71]. As such, extrapolations from city and county bans to state or federal bans should be done with caution, just as results from countries other than the US may not apply to the US setting. In addition, most of the studies are from the US and may have limited applicability in other countries.

All of these factors make direct comparisons of study findings or the pooling of study results infeasible. As such, our scoping review presents an overview of what is known so far and the challenges that researchers need to address. Finally, only one study team member screened titles and abstracts, which may introduce bias into the study selection process.

## Conclusions

Our scoping review outlined the current literature on the effects of flavoured ENDS restrictions. Ultimately, more research is needed to determine the potential effects of flavoured ENDS restrictions on nicotine and tobacco product use behaviours. With numerous US states having implemented bans in recent years, the literature on the effects of these restrictions must be regularly evaluated. Syntheses, such as this paper, provide useful information for both researchers and policymakers in planning evaluations and ensuring that policy decisions are made using the best available evidence. New evaluations of flavour restrictions can fill existing gaps in the literature identified here by considering the balance of effects on youth and adult initiation and cessation of ENDS and combustible cigarettes and by evaluating restrictions in countries beyond the US. As more research is conducted, these effects and additional information on internet sales, do-it-yourself modifications, and illegal marketplaces will provide essential information for a robust consideration of the benefits and harms to public health of flavoured ENDS restrictions.

## Supplementary Information

**Additional file 1.** Appendices 1–7

## Data Availability

The datasets used and/or analyzed during the current study are available from the corresponding author upon reasonable request.

## Abbreviations

CA: California
DCE: Discrete Choice Experiment
DIY: Do-It-Yourself
ENDS: Electronic Nicotine Delivery Systems
FDA: Food and Drug Administration
GRADE: Grading of Recommendations, Assessment, Development and Evaluation
MA: Massachusetts
PRISMA-Sc: Preferred Reporting Items for Systematic reviews and Meta-Analyses Extension for Scoping Reviews
SD: San Diego
SF: San Francisco
SJ: San Jose
US: United States of America
