# Supplementary material for: The actual and anticipated effects of restrictions on flavoured electronic nicotine delivery systems: a scoping review
Source: BMC Public Health. 2022 Nov 19;22:2128. doi: 10.1186/s12889-022-14440-x (PMC9675183; doi:10.1186/s12889-022-14440-x)
Supplement: Supplementary file 1 — Additional file 1. Appendices 1–7 [file 12889_2022_14440_MOESM1_ESM.docx]

**Appendix 1:** Scoping Review Protocol

**Review questions:**

What are the effects (actual or hypothetical/experimental) of bans that include, but are not necessarily limited to, flavoured electronic nicotine delivery systems (ENDS) on tobacco use behaviour and sales?

What compliance concerns have arisen with such bans?

**Databases:** Medline and Scopus

**Search Strategy:** See pg. 5

**Domains being studied:**

Electronic Nicotine Delivery Systems (ENDS) come in many flavours. The wide variety of flavours is thought to appeal to children making them more likely to initiate ENDS use, thereby making flavours a driving factor in the youth vaping epidemic. As a result, there is growing concern that the widespread availability of flavours may lead a new generation to nicotine addiction when youth tobacco use rates are at an all-time low. In particular, flavoured ENDS may contain toxic chemicals that are harmful to health or may act as a gateway to the use of more dangerous tobacco products, namely cigarettes.

In 2018, the US Food and Drug Administration (FDA) published an advanced notice of proposed rulemaking, requesting stakeholders to share data, research and information that could inform the FDA's thinking related to flavours' role in the initiation, use and cessation of tobacco products. Although bans of flavoured and unflavoured ENDS have been increasingly common, limited research exists synthesizing their effects. While bans may successfully reduce youth initiation with ENDS, they may also reduce the benefits of potential harm reduction as fewer current smokers may switch to ENDS. Additionally, those who currently use flavoured ENDS may switch to tobacco flavoured ENDS or even non-flavoured cigarettes due to the ban. Bans may also lead to the rise of illicit products or do-it-yourself flavouring of ENDS which may come with new health risks.

Our scoping review will examine the effects of implemented bans on tobacco use behaviours, sales and industry compliance. In addition, we will review studies of hypothetical bans to present the expected effects of bans in jurisdictions that have not yet implemented them at the time of the study.

**Participants:**

We focus this review on the effects of implemented or hypothetical bans of flavoured ENDS. As such, no limits on study participants will be used, as bans would apply to the whole population. Studies of specific subgroups of interest, such as youth or young adults, and those that evaluate impacts on the general public will be included.

**Interventions:**

We will consider four types of interventions:

1) implemented bans exclusively on flavour ENDS – these studies will report on the effects of a ban on flavours only among ENDS. The ban must apply to only flavoured ENDS, and some flavours may be excluded (such as the FDA's proposal to exclude tobacco and menthol flavours).

2) implemented bans that include flavoured ENDS and other tobacco products or non-flavoured ENDS – these studies will report on the effects of a flavour ban that includes ENDS. The ban may apply to other flavoured tobacco products or all ENDS.

3) hypothetical bans exclusively of flavoured ENDS – participants will be asked how they would respond to a ban on flavours only among ENDS in these studies. These studies may include surveys, interviews, and experimental marketplaces. The ban must apply to only flavoured ENDS; some flavours may be excluded.

4) hypothetical bans that include flavoured ENDS and other tobacco products or non-flavoured ENDS – participants will be asked how they would respond to a flavour ban that includes ENDS in these studies. These studies may include surveys, interviews, and experimental marketplaces. The ban may apply to other flavoured tobacco products or all ENDS.

**Comparators:**

We will examine the effects of flavoured ENDS bans across tobacco product types and subpopulations.

Tobacco product types: Flavoured ENDS users, ENDS users, cigarette users, non-cigarette and non-ENDS users (cigars, smokeless, heated tobacco, etc.).

Subpopulations: age (youth, young adults, adults); race/ethnicity; socioeconomic status; use intensity.

**Types of studies to be included:**

We will include observational studies reporting individual tobacco use behaviours (initiation, cessation, or product switching), product sales, or compliance with the ban. Studies must report on the effects of an implemented flavoured ENDS ban or ask participants what the impact of a hypothetical flavoured ENDS ban would be on their behaviour.

We will exclude animal studies, clinical studies, modelling studies, policy studies, reviews, opinion pieces, and non-peer-reviewed journal articles. Studies that examine the effects of bans that exclude some establishments, such as tobacco bars, vape shops, or over 21 establishments, will be excluded as the effects of such regulations would not be comparable to a ban on all retailers. Finally, studies published in a language other than English will not be included in the review.

**Context:**

Product bans are becoming an increasingly common tool of regulators to address the rapidly ever-changing tobacco marketplace. While bans may successfully reduce youth initiation with ENDS, they may also reduce the benefits of potential harm reduction as fewer current smokers may switch to ENDS. Additionally, those who currently use flavoured ENDS may switch to tobacco flavoured ENDS or even non-flavoured cigarettes due to the ban. Bans may also lead to the rise of illicit products or do-it-yourself flavouring of ENDS which may come with new health risks.

Various jurisdictions, both domestically in the US and internationally, have used bans to limit access to flavoured ENDS. Additionally, tobacco researchers often ask current smokers how they would change their smoking behaviour if certain products were no longer available (using surveys, discrete choice experiments, or experimental tobacco marketplaces, among other study designs). We plan to review both of these studies to gain a broader understanding of the effects of a ban on flavoured ENDS. These findings will help to inform FDA policymaking.

**Main outcomes:**

Our outcomes of interest are divided into four types:

1. Public opinion – we will abstract information from studies on public support for or opposing flavoured ENDS restrictions by tobacco use status.
2. Tobacco use behaviours – we will abstract information from studies on the use of tobacco products, including but not limited to ENDS. We will examine behaviours such as initiation, cessation and product switching resulting from the ban.
3. Product sales – we will abstract information from studies on the change in tobacco product sales following the ban. We will include changes to flavoured ENDS sales, which should decrease to zero following a ban unless certain products are exempted, and changes in sales of other tobacco products which people might switch to as a result of the ban.
4. Ban compliance – we will abstract information on how the tobacco industry, tobacco retailers, and product users comply with the ban. We will include outcomes such as the development of novel replacement products; the use of do-it-yourself flavouring kits; the import, export and/or continued sale of banned products; or crossing jurisdictional boundaries to purchase tobacco products.

**Data extraction:**

Peer-reviewed studies will be included if they empirically consider the effects of an implemented or hypothetical ban on flavoured ENDS. Flavour bans in question do not need to include all flavours. Additionally, studies that include broader bans on products other than ENDS or non-flavoured products will be included if flavoured ENDS are included in the ban.

Non-peer reviewed or empirical studies, as well as studies published in languages other than English, will be excluded. Studies that present an opinion on a flavour ban or do not report results specific to individual behaviour, sales, or compliance will be excluded. Bans that exempt specific locations, such as tobacco bars, vape shops, or over 21 establishments, will be excluded as the effects of such regulations would not be comparable to a ban on all retailers.

We will use the systematic review software DistillerSR to manage the screening of studies. Titles and abstracts will be reviewed separately by two team members. Discrepancies will be resolved by consensus. Two team members will conduct the full-text screening, and reasons for exclusion will be recorded. The primary study team will resolve uncertainty over inclusion at the full-text stage.

A data abstraction template will be developed. Two team members will split the abstracted relevant information from the selected studies. Each team member's abstraction sample will be reviewed for quality control. Abstracted information will include publication-specific details (authors, date, journal, citation), ban type (see data synthesis), study theme (see data synthesis), sample size, age group, location, ban implementation date, list of all banned products, the data source and study method, main outcome types (initiation, cessation, sales, product availability, etc.), results, and study limitations.

**Strategy for data synthesis:**

Following abstraction, the articles will be categorized by type of ban: 1) implemented ban exclusively of flavoured ENDS; 2) implemented a ban that includes flavoured ENDS; 3) hypothetical ban exclusively of flavoured ENDS; 4) hypothetical ban that includes flavoured ENDS. Within each category, the studies will be ordered by theme when applicable: 1) public opinion; 2) individual behaviours (or individual intentions for hypothetical studies); 3) product sales; 4) ban compliance. If applicable, meta-analysis will be used to combine results within ban categories and themes. Summaries of the implications of study findings for policymaking will be presented.

**Subgroups:**

When applicable, comparisons and data pooling will be presented by the subgroups listed in 21. Comparators/controls.

**Deviations from the protocol:**

- Due to study team limitations, only one team member screened studies and abstracted data.
- The search term was further amended following the completion of the protocol and again when key studies were identified as missing and two additional databases were added (see Appendix 3).
- The study team decided at a later point to include studies of restrictions that exempt certain types of retailers.
- The study team added the additional exclusion criteria that required countries to have a legal ENDS market that included flavoured products before the adoption of a flavour restriction.
- Public opinion studies were removed as an outcome of interest in this study given that public opinion does not play a direct role in FDA policymaking and the overall length of the review.
- Quality assessment of the main findings of the review based on the Grading of Recommendations, Assessment, Development and Evaluation approach was added.
- The protocol specifies PICO characteristics. However, PECO (population, exposure, comparator, outcome) characteristics is more suitable to the studies included. As such, the manuscript text refers to exposure to a hypothetical or implemented restriction.

**Search strategy:**

The preliminary search strategy is composed of seven concepts

| Concept 1 – E-Cigarettes | Concept 2 - Flavors | Concept 5 – Ban/Restriction | Concept 6 – Compliance | Concept 7 – Non-ENDS Effects |
| --- | --- | --- | --- | --- |
| E-cigarette*  Electronic cigarette*  Ecigarette  E-cig  Ecig  Novel nicotine delivery products  Electronic nicotine delivery system  ENDS  Nicotine vaping product  Novel nicotine  Vaping  Vape  Vapor  Vaporizer  E-liquid  E-juice  Juul  “Vaping”[MeSH]  “Electronic Nicotine Delivery Systems”[MeSH]  “Nicotine”[MeSH] | Flavor*  Flavour*  Additive*  Mint  Menthol  Fruit  Clove  Cinnamon  Cinnamaldehyde  Sweet  Spicy  Beverage  Desserts  Candy  Cool*  "Flavoring agents"[MeSH] | Ban  Bans  Banning  Restrict*  Sales restriction  Sales restrictions  Product standard  Product standards  Policy  Legislation  Law*  provision  Prohibi*  Regulat*  Jurisprudence  "Smoking/legislation and jurisprudence" [MeSH Terms] | Implement*  Evade  Evasion  Enforce*  Comply  Compliance  Black market  Adherence  Illicit  Illegal  Contraband | Substitut*  Switch*  Transition |

**Appendix 2:** PRISMA Scoping Review Checklist

| **SECTION** | **ITEM** | **PRISMA-ScR CHECKLIST ITEM** | **REPORTED ON PAGE #** |
| --- | --- | --- | --- |
| **TITLE** | | | |
| Title | 1 | Identify the report as a scoping review. | 1 |
| **ABSTRACT** | | | |
| Structured summary | 2 | Provide a structured summary that includes (as applicable): background, objectives, eligibility criteria, sources of evidence, charting methods, results, and conclusions that relate to the review questions and objectives. | 2 |
| **INTRODUCTION** | | | |
| Rationale | 3 | Describe the rationale for the review in the context of what is already known. Explain why the review questions/objectives lend themselves to a scoping review approach. | 4 |
| Objectives | 4 | Provide an explicit statement of the questions and objectives being addressed with reference to their key elements (e.g., population or participants, concepts, and context) or other relevant key elements used to conceptualize the review questions and/or objectives. | 4 |
| **METHODS** | | | |
| Protocol and registration | 5 | Indicate whether a review protocol exists; state if and where it can be accessed (e.g., a Web address); and if available, provide registration information, including the registration number. | 5; Appendix 1 |
| Eligibility criteria | 6 | Specify characteristics of the sources of evidence used as eligibility criteria (e.g., years considered, language, and publication status), and provide a rationale. | 5-6 |
| Information sources* | 7 | Describe all information sources in the search (e.g., databases with dates of coverage and contact with authors to identify additional sources), as well as the date the most recent search was executed. | 5 |
| Search | 8 | Present the full electronic search strategy for at least 1 database, including any limits used, such that it could be repeated. | 5; Appendix 3 |
| Selection of sources of evidence† | 9 | State the process for selecting sources of evidence (i.e., screening and eligibility) included in the scoping review. | 5-6 |
| Data charting process‡ | 10 | Describe the methods of charting data from the included sources of evidence (e.g., calibrated forms or forms that have been tested by the team before their use, and whether data charting was done independently or in duplicate) and any processes for obtaining and confirming data from investigators. | 6 |
| Data items | 11 | List and define all variables for which data were sought and any assumptions and simplifications made. | 6 |
| Critical appraisal of individual sources of evidence§ | 12 | If done, provide a rationale for conducting a critical appraisal of included sources of evidence; describe the methods used and how this information was used in any data synthesis (if appropriate). | NA |
| Synthesis of results | 13 | Describe the methods of handling and summarizing the data that were charted. | 6 |
| **RESULTS** | | | |
| Selection of sources of evidence | 14 | Give numbers of sources of evidence screened, assessed for eligibility, and included in the review, with reasons for exclusions at each stage, ideally using a flow diagram. | 7; Figure 1 |
| Characteristics of sources of evidence | 15 | For each source of evidence, present characteristics for which data were charted and provide the citations. | Appendix 4:6 |
| Critical appraisal within sources of evidence | 16 | If done, present data on critical appraisal of included sources of evidence (see item 12). | NA |
| Results of individual sources of evidence | 17 | For each included source of evidence, present the relevant data that were charted that relate to the review questions and objectives. | 7-18; Appendix 4:6 |
| Synthesis of results | 18 | Summarize and/or present the charting results as they relate to the review questions and objectives. | 7-18; Table 1:3 |
| **DISCUSSION** | | | |
| Summary of evidence | 19 | Summarize the main results (including an overview of concepts, themes, and types of evidence available), link to the review questions and objectives, and consider the relevance to key groups. | 18-19 |
| Limitations | 20 | Discuss the limitations of the scoping review process. | 20 |
| Conclusions | 21 | Provide a general interpretation of the results with respect to the review questions and objectives, as well as potential implications and/or next steps. | 20 |
| **FUNDING** | | | |
| Funding | 22 | Describe sources of funding for the included sources of evidence, as well as sources of funding for the scoping review. Describe the role of the funders of the scoping review. | 1 |

JBI = Joanna Briggs Institute; PRISMA-ScR = Preferred Reporting Items for Systematic reviews and Meta-Analyses extension for Scoping Reviews.

* Where *sources of evidence* (see second footnote) are compiled from, such as bibliographic databases, social media platforms, and Web sites.

† A more inclusive/heterogeneous term used to account for the different types of evidence or data sources (e.g., quantitative and/or qualitative research, expert opinion, and policy documents) that may be eligible in a scoping review as opposed to only studies. This is not to be confused with *information sources* (see first footnote).

‡ The frameworks by Arksey and O'Malley (6) and Levac and colleagues (7) and the JBI guidance (4, 5) refer to the process of data extraction in a scoping review as data charting*.*

§ The process of systematically examining research evidence to assess its validity, results, and relevance before using it to inform a decision. This term is used for items 12 and 19 instead of "risk of bias" (which is more applicable to systematic reviews of interventions) to include and acknowledge the various sources of evidence that may be used in a scoping review (e.g., quantitative and/or qualitative research, expert opinion, and policy document).

*From:* Tricco AC, Lillie E, Zarin W, O'Brien KK, Colquhoun H, Levac D, et al. PRISMA Extension for Scoping Reviews (PRISMAScR): Checklist and Explanation. Ann Intern Med. 2018;169:467–473. [doi: 10.7326/M18-0850](http://annals.org/aim/fullarticle/2700389/prisma-extension-scoping-reviews-prisma-scr-checklist-explanation).

**Appendix 3:** Study Search in Detail

We conducted a two-phase search. An initial search was conducted on July 24, 2021, with the following search term:

Term 1: ((ban OR restrict* OR 'product AND standard' OR prohibi* OR "smoking/legislation and jurisprudence" [MeSH Terms]) AND ("e-cigarette*" OR 'novel AND nicotine AND delivery AND products' OR 'electronic AND nicotine AND delivery AND system' OR 'nicotine AND vaping AND product' OR vape OR vaping OR 'Electronic Nicotine Delivery Systems' [MeSH Terms] OR Vaping[MeSH Terms]) AND ( flavor* OR flavor* OR mint OR menthol OR clove OR cinnamon OR "flavoring agents" [MeSH]))

Term 2: ALL ( ( ( ban OR restrict* OR 'product AND standard' OR prohibi* ) AND ("e-cigarette*" OR 'novel AND nicotine AND delivery AND products' OR 'electronic AND nicotine AND delivery AND system' OR 'nicotine AND vaping AND product' OR vape OR vaping ) AND ( flavor* OR flavor* OR mint OR menthol OR clove OR cinnamon ) ) )

Term 1 was used to search PubMed and Term 2 for Scopus. Based on the results of that search and keywords from other studies that were not initially identified, the search term was revised to:

Term 3: (ban OR restriction* OR "product standard" OR prohibition OR "smoking/legislation and jurisprudence"[MeSH Terms]) AND ("tobacco" OR "e-cigarette*" OR "novel nicotine delivery product" OR "electronic nicotine delivery system" OR "nicotine vaping product" OR vape OR vaping OR "Electronic Nicotine Delivery Systems"[MeSH Terms] OR Vaping[MeSH Terms]) AND ( flavor* OR flavor* OR mint OR menthol OR clove OR cinnamon OR "flavoring agents"[MeSH])

## Term 4: TI-AB-KW: ( ( ban OR restrict* OR "product standard" OR prohibi* OR "legislation" OR "jurisprudence") AND ("tobacco" OR "e-cigarette*" OR "novel nicotine delivery products" OR "electronic nicotine delivery system" OR "nicotine vaping product" OR vape OR vaping ) AND ( flavor* OR flavor* OR mint OR menthol OR clove OR cinnamon OR "flavoring agents") )

Term 3 was used for updated searches of PubMed and Term 4 for updated searches in Scopus and for primary searches in Embase and Web of Science. The final search was conducted on May 3, 2022.

**Appendix 4:** Detailed Description of GRADE Scoring Methods

We use the Grading of Recommendations, Assessment, Development and Evaluation (GRADE) approach to evaluate the quality of the body of evidence presented in the research on flavour restrictions.^1^ Instead of reviewing individual study quality, GRADE ratings and adjustments are based on factors contributing to the strength or weakness of a body of evidence on a given outcome.

GRADE uses four confidence levels from Very Low to High, reflecting the confidence that an outcome is accurate based on the current literature. The definitions of the grades as stated in the GRADE Handbook are provided in Table 4.1. As all empirical studies were observational, in accordance with GRADE, we set the initial quality of the evidence as low. Findings can then be up or downgraded by one or two levels based on different factors. These factors include study design limitations, inconsistencies in results, indirectness of evidence, imprecision, publications bias, the magnitude of effects, minimal confounding effects, and dose-response gradient.^1,2^ Tables 4.2 and 4.3 reproduced from the GRADE Handbook provide additional details on the consequences of the different factors.

**Table 4.1** Quality of Evidence Grades

| Grade | Definition |
| --- | --- |
| High | We are very confident that the true effect lies close to that of the estimate of the effect. |
| Moderate | We are moderately confident in the effect estimate: The true effect is likely to be close to the estimate of the effect, but there is a possibility that it is substantially different |
| Low | Our confidence in the effect estimate is limited: The true effect may be substantially different from the estimate of the effect. |
| Very Low | We have very little confidence in the effect estimate: The true effect is likely to be substantially different from the estimate of effect |

**Table 4.2** Factors that reduce quality rating

| Factor | Consequence |
| --- | --- |
| Limitations in study design or execution (risk of bias) | **↓**1 or 2 levels |
| Inconsistency of results | **↓**1 or 2 levels |
| Indirectness of evidence | **↓**1 or 2 levels |
| Imprecision | **↓**1 or 2 levels |
| Publication bias | **↓**1 or 2 levels |

**Table 4.3** Factors that increase quality rating

| Factor | Consequence |
| --- | --- |
| Large magnitude of effect | **↑** 1 or 2 levels |
| All plausible confounding would reduce the demonstrated effect or increase the effect if no effect was observed | **↑** 1 level |
| Dose-response gradient | **↑** 1 level |

Based on the included studies, the primary study team identified six primary outcomes of interest: two outcomes relating to sales of tobacco products (reduced sales of ENDS and increased sale of combustible cigarettes), three relating to individual behaviours (reduced use of any tobacco product, reduced use of ENDS, increased use of combustible cigarettes), and one relating to compliance (reduced availability of flavoured ENDS) following the adoption of flavoured ENDS restrictions. The included studies were classified as either contributing to a finding or not. One study team member (CC) then reviewed the study outcomes and methods to identify factors across studies that increase or reduce the quality of the evidence and whether these factors were significant (two point increase or decrease) or minor (one point increase or decrease). We considered all empirical studies in our GRADE assessment. Hypothetical studies were excluded. The scores for each result are reported in manuscript Table 4.

**Appendix 5:** Included Sales Studies

| Author, Year | Study Location | Jurisdictional Level | Restriction Details | Main Study Outcomes | Main Study Findings |
| --- | --- | --- | --- | --- | --- |
| Ali, 2022^3^ | Massachusetts, Washington, New York, and Rhode Island compared to 35 control states | State | The total ban on ENDS in Massachusetts; restriction on all non-tobacco flavoured ENDS in all states | Sales of ENDS | Retail sales data from IRI was used to compare the 4 intervention states to 35 non-intervention states.   Prohibition of all e-cigarette products in Massachusetts was associated with a 94.38% (95% CI, 93.37%-95.23%) reduction in mean 4-week total e-cigarette unit sales from November 3 to December 1, 2019, compared with the control states  Beginning in December 2019, this policy was narrowed to restrict the sale of non–tobacco-flavoured e-cigarettes. It was associated with an 88.91% (95% CI, 83.29%-92.64%) reduction in mean 4-week total e-cigarette sales from December 29, 2019, to December 27, 2020, compared with the control states.  In New York, prohibition of non–tobacco-flavoured e-cigarette sales was associated with a 30.65% (95% CI, 24.08%-36.66%) reduction in mean 4-week total e-cigarette sales from June 14 to December 27, 2020, compared with the control states. In Rhode Island, prohibition of non–tobacco-flavoured e-cigarette sales was associated with a 31.26% (95% CI, 11.94%-46.34%) reduction in mean 4-week total e-cigarette sales from November 3, 2019, to December 27, 2020. Washington's temporary policy from November 3, 2019, to January 26, 2020, was associated with a 25.01% (95% CI, 18.43%-31.05%) reduction in mean 4-week total e-cigarette sales.  The increases in sales of tobacco-flavoured e-cigarettes were approximately 40.52%, 43.08%, and 49.17% of the observed total sales decreases in Washington, New York, and Rhode Island, respectively. |
| Gammon, 2021^4^ | San Francisco, San Jose and San Diego, CA | Local | A citywide ban on all flavoured tobacco products in San Francisco was implemented in January 2019. | Changes in weekly sales of ENDS, cigarettes, and total tobacco sales in all three cities. | Flavoured ENDS - SF: pre-policy average weekly sales of 3,439 units; pre-enforcement average weekly sales of 5,906 units; post-enforcement average weekly sales of 16 units (100% decrease). SJ: pre-policy average weekly sales of 1,932 units; pre-enforcement average weekly sales of 4,405 units; post-enforcement average weekly sales of 5,706 units (195% increase). SD: pre-policy average weekly sales of 2,610 units; pre-enforcement average weekly sales of 5,692 units; post-enforcement average weekly sales of 5,701 units (118% increase).  Change in total tobacco sales - SF: pre-policy average weekly sales of 113,191 units; pre-enforcement average weekly sales of 112,657 units; post-enforcement average weekly sales of 84,970 units (25% decrease). SJ: pre-policy average weekly sales of 202,071 units; pre-enforcement average weekly sales of 198,894 units; post-enforcement average weekly sales of 185,679 units (8% decrease). SD: pre-policy average weekly sales of 206,382 units; pre-enforcement average weekly sales of 189,108 units; post-enforcement average weekly sales of 170,764 units (17% decrease).  Change in total ENDS sales - SF: pre-policy average weekly sales of 4,690 units; pre-enforcement average weekly sales of 8,969 units; post-enforcement average weekly sales of 6,772 units (44% increase). SJ: pre-policy average weekly sales of 2,863 units; pre-enforcement average weekly sales of 5,641 units; post-enforcement average weekly sales of 7,756 (171% increase). SD: pre-policy average weekly sales of 4,085 units; pre-enforcement average weekly sales of 7,552 units; post-enforcement average weekly sales of 8,084 units (98% increase). |
| Katchmar, 2021^5^ | Greater Boston Area (Eastern Massachusetts, Southern New Hampshire, Rhode Island, Windham County in Vermont, and Windham County in Connecticut) | State | Massachusetts statewide ban on all ENDS from September 2019 to December 2019; Massachusetts statewide ban on flavoured ENDS December 2019 | Sales of cigarettes and e-cigarettes | During the week of the Massachusetts ban on e-cigarettes, the USA did not see an immediate change in the level of year-over-year purchasing compared to the previous period (p-value for level change = 0.985). However, the Greater Boston area saw a significant decrease in the level compared to the USA (p-value for level change = 0.029). The use of e-cigarettes decreased faster in the greater Boston area than in the US during the ban period. However, while there was no significant change in the level of year-over-year purchasing in the Greater Boston area, there was an increase in the trend relative to the USA, meaning that e-cigarette consumption was declining less rapidly than during the ban.  After the e-cigarette ban in Massachusetts was implemented, there were no significant changes, compared to the previous period, in either the level or trend of cigarette purchasing in the USA (p-value for level change = 0.143, p-value for trend change = 0.069, Table 3). Likewise, there were no significant changes in the level or trend of cigarette purchasing in the Greater Boston area. |
| Liber, 2021^6^ | Washington, Michigan, Oregon, and Massachusetts vs non-intervention control states | State | Total ban on ENDS in Massachusetts; restriction on all non-tobacco flavoured ENDS in all states | Sales of ENDS and cigarettes; compare sales of young age vs. old age brands of cigarettes | With its *Total Ban*, Massachusetts saw a substantial decline in e-cigarette sales, while states that banned all but tobacco-flavoured e-cigarettes lost nearly half their e-cigarette sales. States that did not pass any state-level e-cigarette policies appear to see smaller reductions in the immediate aftermath of EVALI. Partial Bans in Michigan, Oregon and Washington appear to have had the first-order effect of decreasing all menthol and other flavoured product sales while providing a temporary increase to tobacco-flavoured products. The total ban in Massachusetts cut across all product categories, effectively zeroing them out for 3 months, as sales fell 99% from US$10.7 million in August to US$103 000 in December. Once the temporary Total Ban was replaced with a Partial Ban, only tobacco flavours and hardware sales increased, and they did so at a pace 95% smaller than before the outbreak.  Beginning in the 4 weeks ending 5 October 2019—the first period that included the e-cigarette ban imposed on 24 September—sales volumes for all cigarettes in Massachusetts increased for three consecutive 4-week periods (online supplemental figure 2). Average sales volumes in states with partial bans and states that were not subject to a state ban do not appear to be as visibly affected by the outbreak. The increase in cigarette sales is more pronounced for sales of brands that skew young, while brands that skew old do not show any change in their sales trajectory coinciding with the e-cigarette ban in Massachusetts (figure 3).  The state total ban decreased e-cigarette sales by 234% per month at its maximum value. A state partial ban on flavoured e-cigarette sales has a negative sign but is not statistically significant (p=0.16) (-83%). Sales of hardware (279%) and tobacco flavour refills (149% were significantly lower under a state total ban compared to having no restriction in place. A state partial ban was only significantly associated with decreases in sales of menthol-flavoured refills; however, the effect size was considerable, at a 240% decline for a full 4-week period of a partial ban. However, the partial ban effects could be influenced by MA moving from a full to partial ban.   In Massachusetts, a full 4-week period of a total ban on e-cigarette sales was associated with a significant 4.1% rise in sales of total cigarette sales. This effect seems concentrated among disproportionately young cigarette brands, as those increased by 8.3% per period of a full ban, age- proportionate brands increased a more minor but significant 2.7%, and sales of disproportionately old brands were unaffected. |
| Xu, 2022^7^ – Authors of this study are employees of the ENDS company Juul. | Massachusetts, Washington, and Rhode Island | State | Total ban on ENDS in Massachusetts; restriction on all non-tobacco flavoured ENDS in all other exposure states | Cigarette sales | Using Information Resources Inc (IRI data) data collected between January 1, 2018, and December 30, 2019, they estimated the impact of restrictions on cigarette sales. All model specifications found statistically significant increases in cigarette sales in MA. In the preferred model, cigarette sales increased by 7.5% (95% CI: 6.2%-8.9%). In only one model specification is the increase in sales statistically significant in RI and WA. In the preferred model, the increase in sales for these states was 4.6% (95% CI: -0.7% to 9.8%). The model with state-specific estimates suggests statistically significant effects in MA and WA, with a non-significant increase in RI. Event study results suggest that the underlying parallel trends assumption is likely to hold. |

**Appendix 6:** Included Behaviour Studies

| Author, Year | Study Location | Jurisdictional Level | Hypothetical or Implemented Restriction | Restriction Details | Main Study Outcomes | Age Group | Sample Size | Data Source | Results |
| --- | --- | --- | --- | --- | --- | --- | --- | --- | --- |
| Buckell, 2019^8^ | US | National | Hypothetical | Compared 5 policies: ban all flavours; allow only menthol e-cig; ban all cig flavours; only fruit/sweet e-cig; ban all e-cig flavours | Choices for e-cig or combustible cig under each alternative relative to current US policy (no combustible flavour except menthol, all other products allowed) | Adults | 2031 | Discrete Choice Experiment | All flavours banned (percentage point increase): Combustible choice: 2.7 E-cig choice: -7.9 None of these: 5.2  Menthol flavours allowed (percentage point increase): Combustible choice: 0.6 E-cig choice: -4.7 None of these: 4.2  All combustible flavours banned (percentage point increase): Combustible choice: -5.7 E-cig choice: 3.8 None of these: 1.6  E-cig fruit available (percentage point increase): Combustible choice: -3.5 C-cig choice: 1.3 None of these: 2.2  All e-cig flavours banned (percentage point increase): Combustible choice: 8.3 E-cig choice: -11.1 None of these: 3 |
| Farsalinos, 2013^9^ | US, Europe, Asia, Australia | International | Hypothetical | Flavoured ENDS | Attitudes towards restrictions | Adults | 4514 | Fielded Survey | If flavour variability were limited, 68.9% would find e-cigs less enjoyable; 45.7% would find them more boring; 48.5% would have increased cravings for cigarettes; 39.7% would be less likely to reduce or quit smoking (44.2% among current smokers), and it would make no difference to 6.3%. |
| Freitas-Lemos, 2021^10^ | US | National | Hypothetical | Considered no ban, all vaping product ban, or flavoured vaping product ban | Effects of a vaping ban on purchases in illegal markets, effects of price increases | Adults | 150 | Discrete Choice Experiment | Participants from all three groups were more likely to purchase from the IETM if product availability in the LETM was restricted, i.e., under product bans. more likely to choose the IETM under a complete ban on vaping products (OR = 6.93; p < .001) and a partial ban restricting flavored vaping products (OR = 2.83; p < .001) compared to the no ban condition. exclusive e-cigarette users showed the greatest likelihood to purchase from the IETM in response to policies restricting access to all vaping products (OR = 256.20; p < .001) and flavoured vaping products (OR = 33.52; p < .001) when compared to exclusive smokers.   E-cigarette users were less price-sensitive compared to exclusive cigarette smokers (OR = 0.60; p = .003). Cigarette smokers were more likely to buy vaping products in the IETM as the price of cigarettes in the LETM increased. This finding suggests that increasing cigarette prices would result in cigarette smokers switching from their usual products to vaping products and frequent dual users increasing their consumption of vaping products, even if they were only available illegally. |
| Friedman, 2021^11^ | San Francisco, CA | Local | Implemented | All flavoured tobacco products | Past 30-day cigarette use | Youth | 95843 (over multiple waves) | Youth Risk Behavior Surveillance System Survey 2011-2019 | San Francisco's flavour ban was associated with more than doubled odds of recent smoking among underage high school students relative to concurrent changes in other districts (adjusted odds ratio, 2.24 [95% CI, 1.42-3.53]  This result was robust to adjustment for district-specific time trends (adjusted odds ratio, 2.32 [95% CI, 1.45-3.70]; P < .001) and limiting consideration to California (adjusted odds ratio, 2.01 [95% CI, 1.15-3.51] |
| Gravely, 2022^12^ | US, England, Canada | International | Hypothetical | All flavoured ENDS | Support for a ban; expected behaviour | Adults | 851 current vapers (all current or former smokers) - 302 in Canada, 338 in England, 211 in the US | Fielded Survey | Of the sample, 703 regular vapers provided hypothetical behavioural responses. 28.8% would plan to continue vaping with an available flavour, 28.3% would find a way to get banned flavours, 17.1% would stop vaping and smoke cigarettes instead, 12.9% said that they would stop vaping and not smoke, and 12.9% do not know what they would do. There were no differences seen by age; females were more likely to report that they would find a way to get their preferred flavour or to stop vaping and smoking. Dual users were more likely to switch to exclusive combustible use. |
| Hawkins, 2021^13^ | Various counties in Massachusetts | Local | Implemented | Various levels of a flavour ban | Cigarette use and ENDS use | Youth | 10168 ENDS users and 9988 cigarette users (over various waves) | Massachusetts Youth Health Survey | Increasing implementation of flavoured tobacco product restrictions was associated not with a reduction in the likelihood of cigarette use but with a decrease in the level of cigarette use among users (incidence rate ratio −1.56; 95% CI −2.54 to −0.58).  Flavoured tobacco product restrictions also were associated with a reduction in adolescent e-cigarette use (OR −0.87; 95% CI −1.68 to −0.06) |
| Huh, 2021^14^ | California | State | Hypothetical | Flavoured ENDS | Harm perception of e-cigarettes and intention to purchase/use, given a hypothetical flavour ban | Adults | 276 | Surveyed vape shop customers | SEM results found that - Non-tobacco flavour preference was negatively related to e-cigarette dependence (β=−0.19, p=0.002). Vaping to quit smoking was positively associated with e-cigarette dependence (β=0.13, p=0.029). Those who preferred non-tobacco flavours showed significantly lower intention for continued purchase (β=−0.28, p<0.001) and use of e-cigarettes (β=−0.17, p=0.001) in case of a hypothetical flavour ban. Those who reported vaping to quit indicated greater intention for continued purchase (β=0.10, p=0.016) and use of e-cigarettes (β=0.17, p=0.001) in case of a hypothetical flavour ban. |
| Katchmar, 2021^5^ | Massachusetts | State | Implemented | All ENDS; flavoured ENDS | ENDS use; travel to purchase ENDS | Adults | 36 | Fielded Survey | There was no change in the number of respondents who reported daily electronic cigarette use before and after June 1, 2020.  There was an increase in the number of respondents who indicated that they made trips to other states primarily to purchase e-cigarettes |
| Kenkel, 2020^15^ | US | National | Hypothetical | All ENDS flavours except tobacco and menthol | Utility from e-cigarette use; smoking prevalence | Adults | 1200 | Extension of Pesko, 2016 DCE results | The model predicts a small decline in smokers' e-cigarette use from a 2016 flavour ban (16% to 15%); 71% will smoke, and 13% will use nicotine replacement therapy. |
| Kingsley, 2019^16^ | Lowell and Malden, MA | Local | Implemented | All flavoured tobacco products | Flavoured product availability and youth tobacco product use | Youth | 1229 | Fielded Survey | There were no significant differences in the likelihood that a student initiated with flavoured tobacco from baseline to follow-up between Lowell and Malden.  Difference-in-difference models found a marginally significant difference between the two communities’ change in ever use of any flavoured tobacco product (−6.1%, p=0.07) and a significant difference in change in current use of any flavoured tobacco product (−5.7%, p=0.03).  Difference-in-difference models found significant differences between the two communities’ changes in ever-use (−8.6%, p=0.01) and current-use (−6.2%, p=0.01) of any non-flavoured product. |
| Kingsley, 2021^17^ | Salem, Attleboro, Gloucester, MA | Local | Implemented | All flavoured tobacco products | Initiation of flavoured products, tobacco product use, sources of tobacco products, location of product acquisition; | Youth | 2814 | Fielded Survey | Ever-use and current use of flavoured and nonflavored tobacco increased from baseline to follow-up; current use of flavoured and nonflavored tobacco was significantly smaller in adopting municipalities compared to the comparison (−9.4% [−14.2%, −4.6%] and −6.3% [−10.8%, −1.8%], respectively); DiD results were not statistically significant. There were significantly smaller increases in current use of flavored e-cigarettes Salem(-11.6% (-17.7%, -5.4%)) Attleboro (-7.6% (-12.3%, -2.8%)). Implemented area students less likely to report they knew someone who would buy tobacco for them if asked (−16.5% [−20.8%, −12.3%])  Also did focus groups - only students in EA reported going to another state to buy products. One student in EA talked about tobacco age restrictions in the following way: "It's 18 there [in Rhode Island], 21 in MA. So I just go there." |
| Liu, 2022^18^ | Oakland, CA | Local | Implemented | All flavoured products except in adult-only establishments | Vaping and cigarette use | Youth | NR | Youth Risk Behavior Surveillance System Survey 2015-2019 | Following the sales restriction, in Oakland, high school youth vaping and cigarette use declined between 2017 and 2019. |
| Olson, 2022^19^ | Minneapolis and St. Paul, MN | Local | Implemented | All flavoured tobacco products with over 21 establishments are exempt | Any tobacco use (cigarette cigar, smokeless, e-cig, hookah any use) - defined as saying a user and use of at least 1 of past 30 days | Youth | Youth Tobacco Survey – appx. 4000 per wave Student Survey – appx. 125,000 per wave | Minnesota Student Survey and Minnesota Youth Tobacco Survey | Between 2014 and 2017, the flavour policies were implemented, and the prevalence of any tobacco product use and cigar smoking prevalence did not change significantly in the Twin Cities among youth (any tobacco product: 18.1%e 17.6%; cigars: 6.3%e6.9%; and both not significant); however, the prevalence of e-cigarette use increased in the Twin Cities from 11.1% to 14.9% (þ34.1%, p < .05) (Figure 2). In contrast, prevalence of any tobacco use in ROS increased from 12.4% to 15.7% (þ26.6%, p < .05) owing to increased use of cigars (þ71.3%) and e-cigarettes (þ114.0%) (p < .05). Smokeless tobacco use prevalence decreased in the Twin Cities from 3.6% to 2.2% (p < .05) but did not change in ROS; pipe use prevalence decreased in both geographies (Twin Cities: from 3.6% to 1.1%, p < .05; ROS: from 2.8% to 1.7%, p < .05). No significant changes were detected in either cigarette use prevalence or hookah use prevalence in the Twin Cities or ROS.  Any tobacco product use among youth increased in ROS between 2014 and 2017 but did not appear to change in the Twin Cities (Minneapolis and St. Paul flavour policies were implemented in 2016). During the same period, youth cigar use prevalence significantly increased in ROS but did not change in the Twin Cities. Although statistically significant increases were observed for youth e-cigarette use prevalence in both the Twin Cities and ROS, prevalence increased to a lesser extent in the Twin Cities (34.1%) than in ROS (114%). |
| Pacek, 2019^20^ | US | National | Hypothetical | Three scenarios: 1) nicotine-free; 2) no flavours except tobacco/mint; 3) not customizable | Anticipated EC and CC use under the three hypothetical scenarios | Young adults (18-29) | 240 | Fielded Survey | Nicotine restriction scenarios: Respondents were more likely to report intentions to quit or reduce e-cigarette (EC) versus combustible cigarette (CC) use; were more likely to report intentions to maintain or increase CC use versus EC use. They reported intentions to: Quit - EC: ~34%; CC: ~7% Reduce - EC: ~37%; CC: ~18% Maintain - EC: ~17%; CC: ~26% Increase - EC ~9%; CC ~47%  Flavour restrictions scenario: Respondents were more likely to report intentions to quit or reduce EC versus CC use; were more likely to report intentions to maintain or increase CC use versus EC use. They reported intentions to: Quit - EC: ~12%; CC: ~5% Reduce - EC: ~42%; CC: ~25% Maintain - EC: ~40%; CC: ~54% Increase - EC ~5%; CC ~18%  Device type restrictions scenario: Respondents were more likely to report intentions to quit or reduce EC versus CC use; more likely to report intentions to increase CC use versus EC use. They reported intentions to: Quit - EC: ~12%; CC: ~6% Reduce - EC: ~37%; CC: ~18% Maintain - EC: ~48%; CC: ~55% - not sig dif Increase - EC ~2%; CC ~22% |
| Pesko, 2016^21^ | US | National | Hypothetical | Flavoured ENDS except for mint and tobacco | ENDS selection | Adults | 1200 | Discrete Choice Experiment | Increased flavour availability increased ENDS selection, from 17.5 to 21.9% for younger adults (P< 0.001), but was not associated with a practically or statistically significant increase for older adults. Increased flavour availability increased ENDS selection (P < 0.001) for individuals who have not used vaping devices in the past month but was not associated with a statistically significant increase in ENDS selection for individuals who have. Regardless of cigarette quitting interest, both populations increased the selection of ENDS products when more flavours were available.  ENDS flavours influence young adult smokers. In contrast, older adult smokers are not, which suggests that removing flavour availability could have a relatively minor impact on adult smokers using ENDS (potentially for smoking cessation/reduction) but could have a large impact on the attractiveness of ENDS for adolescents. |
| Posner, 2021^22^ | Atlanta, GA; San Diego, CA; Boston, MA; Oklahoma City, OK; Seattle, WA | Local | Hypothetical | Flavoured ENDS; all ENDS | Support for restrictions and behavioural intentions | Young adults (18-34) | 2159 (550 ENDS users) | Fielded Survey | If a policy was implemented to restrict flavoured vaping products to tobacco flavour only, 39.1% of e-cigarette users indicated being very or somewhat likely to continue using e-cigarettes, and 33.2% of e-cigarette users reported being very or somewhat likely to switch to traditional cigarettes. Also, 14.9% reported being very or somewhat likely to continue using e-cigarettes and switching to traditional cigarettes.  30.5% of e-cigarette users reported being not at all likely to continue to use e-cigarettes, and 45.5% of e-cigarette users reported being not at all likely to switch to traditional cigarettes (17.1% reported being not at all likely to do either).   If all vape product sales were restricted, 39.4% of e-cigarette users said they would be very or somewhat likely to switch to traditional cigarettes. However, 38.9% of e-cigarette users reported being not at all likely to switch to traditional cigarettes. Among e-cigarette users who did not currently use cigarettes: a) 72.2% reported being "not at all likely" (39.4%) or "a little likely" (32.8%) to continue vaping if vape product flavours were restricted; b) 79.8% reported being "not at all likely" (72.7%) or "a little likely" (7.1%) to switch to cigarettes if vape product flavours were restricted; and c) 75.8% reported being "not at all likely" (63.7%) or "a little likely" (12.1%) to switch to cigarettes if all vape products were restricted. |
| Yang, 2020^23^ | San Francisco, CA | Local | Implemented | All flavoured tobacco products | Use of flavoured tobacco products before and after the ban. | Young adults (18-34) | 247 | Fielded survey after policy implementation | Reported reductions in tobacco use: Use of any tobacco product: ages 18-24: −17.7 percentage points (−27.5, −8.0); ages 25-34: −7.6 percentage points (−11.4, −3.7)   Use of any flavoured product: ages 18-24: −11.3 percentage points (–23.6,1.0); ages 25-34:−8.6 percentage points (−14.0, −3.3)   Use of any cigarette product: ages 18-24: 9.7 percentage point increase (−1.3, 20.7); ages 25-34: 0.5 percentage point increase (−5.6, 6.7)   Use of any e-cigarette product: ages 18-24: −9.7 percentage points (−21.6, 2.2); ages 25-34: −9.2 percentage points (−15.4, −3.0)  Use of any flavoured e-cigarette product: ages 18-24:−11.3 percentage points (–22.7, 0.07); ages 25-34: −8.1percentage points (−14.7, −5.0)   Of the 58 exclusive flavoured e-cig users and the 81 poly flavoured users 35 and 53 (respectively) maintained use; 12 and 3 quit any tobacco use; 11 and 25 using any other products. |

**Appendix 7:** Included Compliance Studies

| Author, Year | Study Location | Jurisdictional Level | Restriction Details | Method | Main Study Outcomes | Results |
| --- | --- | --- | --- | --- | --- | --- |
| Amalia, 2020^24^ | India | National | All ENDS | Retail observation | Compliance of retail storefronts across India with the ban on ENDS | Of the 199 retailer storefronts visited, 37 (18.6%) sold ENDS. Kolkata had the highest availability of non-compliant retailers (n=26; 83.9%). The majority of the non-compliant retailers were tobacco retailers (n=35; 94.6%), sold e-cigarettes (n=22; 59.5%), and e-cigarette accessories (n=24; 64.9%). Finally, 90% of stores that sold ENDS were aware of the ban. |
| Amalia, 2020^25^ | India | National | All ENDS | Online purchase attempts | Content analysis of internet e-cig vendors; whether or not they would deliver to New Delhi | Searches were conducted on Nov 26-27, 2019. Searches identified 45 unique websites, 16 of which (35.6%) delivered at least one vaping product to New Delhi. Half of the non-compliant websites were general e-commerce; 10 were retailers from other countries; 75% did not require age verification. All pages were in English.  Half of the sites sold e-liquids; seven sold both nicotine and nicotine-free liquids. Cannabis products were available in 4. Addition information on currency, health claims, and prices provided. |
| Andersen-Rodgers, 2021^26^ | California | Local | Comprehensive (all product) flavour restrictions | Mixed methods: compliance checks, surveys of store owners | Whether stores sold various flavoured products; owners' attitudes toward flavoured tobacco ordinances. | Retail Observation 165 stores with bans; 160 without Compared to stores in matched no-ordinance jurisdictions, a significantly lower proportion of stores in flavour ordinance jurisdictions sold menthol cigarettes (40.6% vs. 95.0%), cigarillos/cigar wraps with explicit flavour names (56.4% vs. 85.0%) and vaping products with explicit flavour names (6.1% vs. 56.9%).  Retailer Poll Of 1703 retail tobacco employees, 36.6% were owners, 39.9% were managers, and 23.5% were clerks. A significantly lower proportion of retail employees in ordinance jurisdictions agreed that flavoured tobacco products are intended to get youth addicted to nicotine (39.4% vs. 48.1%) A significantly lower percentage of retail employees in ordinance jurisdictions: (a) agreed that flavoured tobacco products should only be sold in stores that require customers to be at least 21 to enter (55.2% vs. 61.8%), (b) agreed that flavoured tobacco products appeal to youth (52.1% vs 58.2), and (c) supported banning the sale of flavoured tobacco products (32.1% vs 37.4%). |
| Brock, 2019^27^ | Minnesota | Local | All flavoured tobacco products outside of adult-only establishments | Pre and post retail observations | Availability of flavoured tobacco products in stores | Significantly fewer of the convenience/grocery stores sold flavoured tobacco in Minneapolis (85.4% vs 39.0%) and Saint Paul (97.3% vs 8.1%)  Note: does not separate out ENDS. |
| Gaiha, 2021^28^ | California | Local | Restrict all tobacco flavours | Survey of 602 flavoured JUUL users and 650 other ENDS users ages 15-29 | Percentage of underage users who obtain flavoured Juul and other e-cigs in restricted areas | Underage Juul users - 33.6% report obtaining flavoured Juul from brick and mortar retail, and 11.6% online, from social sources (76.0%); Underage users report obtaining non-Juul e-cigarettes 31.2% of the time from retail stores and 12.7% online, social sources 70.9%  Jurisdictions with restrictions had lower odds of retail purchases (AOR 0.54; 0.36-0.80 - Juul) (AOR 0.48; 0.320-0.72 - non-Juul) Living in a jurisdiction with a ban did not reduce the likelihood of online sales access but did increase the odds of social access (AOR 1.55; 1.02 - 2.35 - Juul) (AOR 194; 1.29 - 2.90 - non-Juul) For under age, social sources are highly significant compared to young adults (AOR 2.19; 1.44 - 3.30 - Juul) (AOR3.07; 2.04-4.64 - Non-Juul) |
| Holmes, 2022^29^ | San Francisco and cities in Alameda County | Local | Various policies are implemented in Alameda from comprehensive, partial and no policy. By the second round of data collection, SF, Alameda, Albany Livermore and Freemont had comprehensive bans, Berkley Hayward, Oakland, and San Leandro had partial bans (exempting menthol or limiting sales only near schools) | Pre and post retail observations | Availability of various flavoured products before and after implementation, and between policy and no policy cities | Overall, retail availability of tobacco decreased significantly in the region. For ENDS, Blu menthol e-cigarette availability fell from 53.3% of stores pre-ban to 6.2% post-ban compared to 64.3% of pre-period no ban stores and 34.3% post-period no ban stores. Difference between two areas statistically significant. Availability of flavoured e-cigarettes, cigalikes, mod/tank devices, and e-liquids was stat sig lower in the ban regions in the post period. |
| Kephart, 2020^30^ | Boston, MA | Local | All flavoured tobacco products (except for mint/menthol) at non-adult establishments | Pre and post retail observations | Availability of flavoured products | At baseline, 88.6% of retailers sold flavoured tobacco products (100% of complete cases). In the complete cases, on average, 19.5 flavoured products were sold, and 58.9% had flavoured product advertising. At follow-up, 14.4% sold flavoured products; an average of 0.39 flavoured products were sold (or 3 per store still selling), and 28% had flavoured product advertising.    E-cigarettes & liquids made up 16.4% of unique flavoured products identified prior to the ban and 11.8% following the ban. The number of e-cig/liquid products available decreased from 1135 to 17.  Of the 51 retailers not in compliance, 72.5% did not know a product was in violation.  Shelf space was most commonly used for non-flavoured tobacco (70.2%) 0r non-tobacco products 13.0%. |
| Kingsley, 2019^16^ | Lowell, MA | Local | Flavoured tobacco products | Retail observations in Lowell and control city of Malden, MA. | Flavour product availability | In Lowell, the number of flavoured products sold per retailer decreased significantly from baseline to follow-up. There was a 70–percentage point decrease in the number of stores where flavoured products were available (from 77.3% to 7.3%, p<0.001). No change in control city. |
| Nali, 2021^31^ | Massachusetts | State | All ENDS products | Online purchase attempts | Availability of online purchases for ENDS shipped to MA | Of the 50 total online ENDS vendors reviewed, orders conducted on 1 October revealed that 38 (76%) were non-compliant, allowing the processing of simulated online purchases after entering a Massachusetts shipping address in the online order. Of the non-compliant vendors, 23 (79.3%) with IP addresses in the US and 15 (71.4%) with IP addresses in Canada allowed simulated orders to Massachusetts. Of the 12 compliant stores that did not permit simulated orders to Massachusetts, 6 had IP server locations within the US, and the other six were in Canada.   Further simulated purchases on 9 October 2019 found that a total of 15 stores were compliant, which included three stores that changed their processes to restrict shipments to Massachusetts addresses, with two of these having IP addresses from the United States and a third with an IP address from Canada. The final set of simulated purchases was conducted on 7 November 2019 and found that 14 stores (7 in the US and 7 in Canada) restricted shipments to a Massachusetts address, including one store that returned to a non-compliant status. |
| Vyas, 2021^32^ | San Francico, CA | Local | Flavours in all tobacco products, including menthol | Retail observation | Level of compliance with restrictions | Prior to enforcement, but 6 months after the successful referendum, 17% of retailers had no flavours available. Compliance peaked at 100% shortly before and after enforcement started in April 2019. From Jul to Dec 2019 compliance ranged from 77-80%. The average compliance from Jan to Dec 2019 was 80% |

**References**

1. Schünemann H, Brożek J, Guyatt G, Oxman A. GRADE handbook for grading quality of evidence and strength of recommendations. Updated October 2013. The GRADE Working Group, 2013. In:2019.

2. Rogers T, Brown EM, Siegel-Reamer L, et al. A Comprehensive Qualitative Review of Studies Evaluating the Impact of Local US Laws Restricting the Sale of Flavored and Menthol Tobacco Products. *Nicotine Tob Res.* 2021.

3. Ali FRM, Vallone D, Seaman EL, et al. Evaluation of Statewide Restrictions on Flavored e-Cigarette Sales in the US From 2014 to 2020. *JAMA Netw Open.* 2022;5(2):e2147813-e2147813.

4. Gammon DG, Rogers T, Gaber J, et al. Implementation of a comprehensive flavoured tobacco product sales restriction and retail tobacco sales. *Tob Control.* 2021.

5. Katchmar A, Gunawan A, Siegel M. Effect of Massachusetts House Bill No. 4196 on electronic cigarette use: a mixed-methods study. *Harm Reduct J.* 2021;18(1):50.

6. Liber AC, Cahn Z, Diaz MC, Donovan E, Vallone D, Schillo B. The EVALI outbreak and tobacco sales in the USA, 2014–2020. *Tob Control.* 2021:tobaccocontrol-2021-056807.

7. Xu Y, Jiang L, Prakash S, Chen T. The Impact of Banning Electronic Nicotine Delivery Systems on Combustible Cigarette Sales: Evidence From US State-Level Policies. *Value Health.* 2022.

8. Buckell J, Marti J, Sindelar JL. Should flavours be banned in cigarettes and e-cigarettes? Evidence on adult smokers and recent quitters from a discrete choice experiment. *Tob Control.* 2018.

9. Farsalinos KE, Romagna G, Tsiapras D, Kyrzopoulos S, Spyrou A, Voudris V. Impact of flavour variability on electronic cigarette use experience: an internet survey. *Int J Environ Res Public Health.* 2013;10(12):7272-7282.

10. Freitas-Lemos R, Stein JS, Tegge AN, et al. The Illegal Experimental Tobacco Marketplace I: Effects of VapingProduct Bans. *Nicotine Tob Res.* 2021.

11. Friedman AS. A Difference-in-Differences Analysis of Youth Smoking and a Ban on Sales of Flavored Tobacco Products in San Francisco, California. *JAMA Pediatr.* 2021;175(8):863-865.

12. Gravely S, Smith DM, Liber AC, et al. Responses to potential nicotine vaping product flavor restrictions among regular vapers using non-tobacco flavors: Findings from the 2020 ITC Smoking and Vaping Survey in Canada, England and the United States. *Addict Behav.* 2022;125:107152.

13. Hawkins SS, Kruzik C, O'Brien M, Levine Coley R. Flavoured tobacco product restrictions in Massachusetts associated with reductions in adolescent cigarette and e-cigarette use. *Tob Control.* 2021.

14. Huh J, Yu S, Galimov A, et al. Hypothetical flavour ban and intention to vape among vape shop customers: the role of flavour preference and e-cigarette dependence. *Tob Control.* 2021.

15. Kenkel DS, Peng S, Pesko MF, Wang H. Mostly harmless regulation? Electronic cigarettes, public policy, and consumer welfare. *Health economics.* 2020;29(11):1364-1377.

16. Kingsley M, Setodji CM, Pane JD, et al. Short-Term Impact of a Flavored Tobacco Restriction: Changes in Youth Tobacco Use in a Massachusetts Community. *Am J Prev Med.* 2019;57(6):741-748.

17. Kingsley M, Setodji CM, Pane JD, et al. Longer-Term Impact of the Flavored Tobacco Restriction in Two Massachusetts Communities: A Mixed-Methods Study. *Nicotine Tob Res.* 2021;23(11):1928-1935.

18. Liu J, Hartman L, Tan ASL, Winickoff JP. Youth tobacco use before and after flavoured tobacco sales restrictions in Oakland, California and San Francisco, California. *Tob Control.* 2022:tobaccocontrol-2021-057135.

19. Olson LT, Coats EM, Rogers T, et al. Youth Tobacco Use Before and After Local Sales Restrictions on Flavored and Menthol Tobacco Products in Minnesota. *J Adolesc Health.* 2022.

20. Pacek LR, Rass O, Sweitzer MM, Oliver JA, McClernon FJ. Young adult dual combusted cigarette and e-cigarette users' anticipated responses to hypothetical e-cigarette market restrictions. *Subst Use Misuse.* 2019;54(12):2033-2042.

21. Pesko MF, Kenkel DS, Wang H, Hughes JM. The effect of potential electronic nicotine delivery system regulations on nicotine product selection. *Addiction.* 2016;111(4):734-744.

22. Posner H, Romm K, Henriksen L, Bernat D, Berg CJ. Reactions to sales restrictions on flavored vape products or all vape products among young adults in the US. *Nicotine Tob Res.* 2021.

23. Yang Y, Lindblom EN, Salloum RG, Ward KD. The impact of a comprehensive tobacco product flavor ban in San Francisco among young adults. *Addict Behav Rep.* 2020;11:100273.

24. Amalia B, Kapoor S, Sharma R, Singh RJ. E-cigarette retailer storefront availability following a nationwide prohibition of e-cigarettes in India: A multicentric compliance assessment. *Tob Prev Cessat.* 2020;6:42.

25. Amalia B, Kapoor S, Sharma R, Fu M, Fernández E, Rana JS. Online sales compliance with the electronic cigarettes ban in India: a content analysis. *Int J Public Health.* 2020;65(8):1497-1505.

26. Andersen-Rodgers E, Zhang X, Vuong TD, et al. Are California's Local Flavored Tobacco Sales Restrictions Effective in Reducing the Retail Availability of Flavored Tobacco Products? A Multicomponent Evaluation. *Eval Rev.* 2021;45(3-4):134-165.

27. Brock B, Carlson SC, Leizinger A, D'Silva J, Matter CM, Schillo BA. A tale of two cities: exploring the retail impact of flavoured tobacco restrictions in the twin cities of Minneapolis and Saint Paul, Minnesota. *Tob Control.* 2019;28(2):176-180.

28. Gaiha SM, Henriksen L, Halpern-Felsher B, et al. Sources of flavoured e-cigarettes among California youth and young adults: associations with local flavoured tobacco sales restrictions. *Tob Control.* 2021.

29. Holmes LM, Lempert LK, Ling PM. Flavored Tobacco Sales Restrictions Reduce Tobacco Product Availability and Retailer Advertising. *Int J Environ Res Public Health.* 2022;19(6).

30. Kephart L, Setodji C, Pane J, et al. Evaluating tobacco retailer experience and compliance with a flavoured tobacco product restriction in Boston, Massachusetts: impact on product availability, advertisement and consumer demand. *Tob Control.* 2020;29(e1):e71-e77.

31. Nali MC, Purushothaman V, Xu Q, Cuomo RE, Mackey TK. Characterizing and assessing compliance of online vendors to the state of Massachusetts ENDS product sales ban. *Tob Induc Dis.* 2021;19:05.

32. Vyas P, Ling P, Gordon B, et al. Compliance with San Francisco's flavoured tobacco sales prohibition. *Tob Control.* 2021;30(2):227-230.
